# Navigating Dementia Care in Northern Ontario: Exploring Health Professional Experience, Barriers and Facilitators

**DOI:** 10.1002/alz.092934

**Published:** 2025-01-09

**Authors:** Lucy Shrestha, Hom Lal Shrestha

**Affiliations:** ^1^ Laurentian University, Sudbury, ON Canada

## Abstract

**Background:**

Dementia is the seventh leading cause of death worldwide, impacting cognitive abilities and memory loss among aging populations. With the increasing prevalence of dementia, it is crucial to understand the distinct barriers and possibilities in providing care. Although there have been significant studies on dementia care, there is a lack of research that primarily focuses on the perspectives and worldviews of healthcare professionals, especially in the northern and rural regions of Ontario.

**Objectives:**

The objective of this study is to identify the essential components required to support healthcare professionals in caring for people living with dementia (PLWD) in Northern Ontario.

**Methods:**

Through an appreciative inquiry framework, data collection includes semi‐structured interviews with healthcare professionals caring for PLWD. A literature review was conducted to understand what has been published on the perspectives of health professionals in dementia care. A comprehensive search in Google Scholar, PubMed, and the National Library of Medicine was conducted through peer‐reviewed research articles published between 2014 and 2023 that focused on quantitative, qualitative, and mixed methods.

**Result:**

The peer‐reviewed articles serve as a basis for comprehending dementia care focusing on the experiences and perceptions of healthcare professionals on dementia care within a Canadian context. These articles address several aspects, such as the challenges, barriers, facilitators, and recommendations for enhancing the quality of care provided to PLWD. Furthermore, they discussed the possibility of enhancing the accessibility of healthcare facilities, researching to gain a deeper understanding of providing higher‐quality care and emphasizing the importance of ongoing support and training for healthcare workers specializing in dementia care.

**Discussion:**

The worldview of health professionals in dementia care aims to reform and enhance health professionals for PLWD to improve care, support, and inform future practice in this area. The findings are broadly shared and contribute to informing future policymaking by including specialized educational materials, training programs designed to raise awareness and practice, encourage advocacy, improve comfort levels, and ultimately enhance patient care for those with dementia. Furthermore, it could explore the possibility of implementing community‐driven initiatives and assistance programs for PLWD and their families residing in rural regions.

